# Bolus-Infusion Delays of Alteplase during Thrombolysis in Acute Ischaemic Stroke and Functional Outcome at 3 Months

**DOI:** 10.1155/2014/358640

**Published:** 2014-04-30

**Authors:** Paul Acheampong, Margaret T. May, Gary A. Ford, Anand K. Dixit

**Affiliations:** ^1^Acute Stroke Service, Royal Victoria Infirmary, Newcastle upon Tyne NE1 4LP, UK; ^2^School of Social and Community Medicine, University of Bristol, Bristol BS8 2PS, UK; ^3^Institute for Ageing and Health, Newcastle University, Newcastle upon Tyne NE2 4HH, UK

## Abstract

*Background*. The efficacy of alteplase in acute ischaemic stroke (AIS) is highly time dependent. Hence, alteplase is administered as soon as possible with a bolus followed by an infusion. Delays between bolus and infusion may occur, but the extent of these delays and the impact on outcome are unclear. *Aims*. We investigated the extent of bolus-infusion delays and the relationship between delays and stroke outcome. *Method*. We reviewed medical records of 276 patients who received alteplase for AIS at our centre between April, 2008, and June, 2013. Complete demographic and clinical data including 3-month modified Rankin Score (mRS) from 229 patients were analysed comparing delays of 0–8 and >8 minutes. *Results*. Overall mean (SD) bolus-infusion delay was 9 (7) minutes. Baseline characteristics were similar apart from more severe strokes in delays >8 minutes. Three-month outcomes were not significantly different although delays >8 minutes had lower functional independence rate (mRS 0-1: 23.1% versus 28.1%; adjusted OR 1.2 (95% CI 0.6 to 2.4, *P* = 0.68)) and higher mortality rate (18% versus 11%, OR 1.0, 95% CI 0.6 to 1.7, *P* = 0.95). *Conclusions*. In this single centre series, bolus-infusion delays of alteplase in AIS were common and no effect of bolus-infusion delays on independence and mortality was found.

## 1. Introduction

Alteplase is licensed for use in acute ischaemic stroke (AIS). Its efficacy in improving stroke outcome depends on onset to treatment time (OTT) [1]. Alteplase has a short plasma half-life of about 4 minutes [2] and is administered at a dose of 0.9 mg/kg body weight with 10% of the total dose given as a bolus over 1-2 minutes and the remainder infused over an hour. This regimen has been demonstrated to achieve functional improvement with a low risk of intracranial haemorrhage [3].

None of the trials of alteplase in AIS reported the extent of bolus-infusion delays. Indeed, there are very limited data on the pharmacokinetic profile of alteplase as used in AIS [4, 5]. Considering the short half-life of alteplase, significant bolus-infusion delays may result in delayed or suboptimum steady state kinetics which could adversely influence outcome.

We investigated the extent of bolus-infusion delays of alteplase in AIS in a single centre and its relationship with functional outcome at 3 months.

## 2. Materials and Methods

We retrospectively reviewed medical records of patients treated with alteplase within 4.5 h after AIS at Newcastle upon Tyne Hospitals Trust from April 2008 to June 2013. Data on baseline demographics and relevant clinical parameters, functional outcome based on modified Rankin Scale (mRS) score at 3 months, and time lapse between bolus and infusion doses of alteplase were retrieved.

Bolus-infusion delays have been reported per typical half-lives (4 minutes) and further categorized as 0–8 and >8 minutes for inference analysis based on the likelihood of significant decline in plasma concentration of alteplase after a bolus-infusion delay of 2 half-lives [5]. Demographic and clinical data have been reported as descriptive summaries with inference statistics using independent *t*-test and chi-square (*χ* ^2^) test.

We analysed the relationship between independent functional outcome (mRS 0-1 and 0–2) at 3 months and bolus-infusion delays of 0–8 and >8 minutes using multivariable logistic regression (MLR) adjusting for age, sex, blood pressure, atrial fibrillation and hyperlipidaemia status, antiplatelet use, prestroke mRS, Oxfordshire Community Stroke Project classification, prethrombolysis NIHSS, and OTT. Patients who received at least two-thirds of the total recommended intravenous dose of alteplase without intra-arterial or thrombectomy interventions were included in the analysis based on pharmacokinetic models which predict attainment of ≥90% of the potential peak plasma concentration from full dose alteplase assuming a bolus injection time of 1 minute and bolus-infusion delay of 5–15 minutes [5]. IBM SPSS version 20 (New York, USA) and Stata statistical software version 12 (College Station, Texas, USA) were used for the analysis.

## 3. Results

Medical records of 276 patients with AIS treated with intravenous thrombolysis were reviewed. A total of 229 patients were included in the analysis after the exclusion of 47 patients with incomplete data involving admission glucose, infusion start time and/or mRS at 3 months (*n* = 24), endovascular treatment including intra-arterial thrombolysis and thrombectomy (*n* = 17), and administration of less than two-thirds of total dose of alteplase mainly due to early termination of alteplase infusion (*n* = 6). Demographic and clinical characteristics of excluded patients were similar to those included in the analysis.

Baseline characteristics were similar between the groups with bolus-infusion delays of 0–8 and >8 minutes except for the latter group that had more severe strokes as determined by prethrombolysis NIHSS (Table 1). Bolus-infusion delays of 0–4, 5–8, 9–12, and >12 minutes occurred in 20%, 33%, 25%, and 22% of patients, respectively, with an overall mean (SD) delay of 9 (7) minutes.

**Figure 1:**
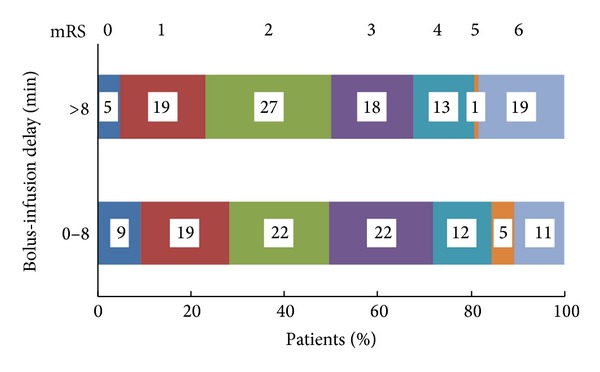
Bolus-infusion delays during treatment with intravenous alteplase within 4.5 h and outcome at 3 months after stroke. mRS-modified Rankin Scale.

Unadjusted functional independence at 3 months was similar among the two groups (mRS 0–2: 50% versus 50%, continuity correction *χ* ^2^  test = 0, unadjusted OR 1.0, 95% CI 0.6 to 1.7, and *P* = 0.95. mRS 0-1: 28.1% versus 23.1% for delays of 0–8 and >8 minutes, resp., continuity correction *χ* ^2^  test = 0.495, unadjusted OR 0.8, 95% CI 0.4 to 1.4, and *P* = 0.39). Adjusted OR for an independent functional outcome for delays >8 minutes was marginally significant at 2.1 (95% CI 1.0 to 4.1, *P* = 0.04) based on mRS 0–2 but insignificant using mRS 0-1 at 1.2 (95% CI 0.6 to 2.4, *P* = 0.68). As expected, independent functional outcome at 3 months was adversely affected by higher prethrombolysis NIHSS score. Compared with NIHSS 0–9, unadjusted OR for good functional outcome were 0.28 (95% CI 0.14 to 0.56, *P* < 0.001) and 0.07 (95% CI 0.02 to 0.25, *P* < 0.001) for NIHSS 10–19 and >19, and adjusted OR were 0.46 (95% CI 0.18 to 1.19, *P* = 0.11) and 0.10 (95% CI 0.02 to 0.42, *P* = 0.002), respectively. The mortality rate at 3 months was nonsignificantly higher in the group with delays >8 minutes (11% versus 18%, difference 7.8, CI −2.2 to 17.8, continuity correction *χ* ^2^  test = 2.2, and *P* = 0.138) (Figure 1).

## 4. Discussion

In this single centre study, we observed bolus-infusion delays of >8 minutes in 47% of AIS patients treated with intravenous alteplase. Compared to those with delays >8 minutes, bolus- infusion delays of ≤8 minutes were associated with nonsignificantly higher independent functional outcome and lower mortality rates after 3 months. Our findings are similar to those reported in abstract form in a smaller series of 120 patients where no difference in functional outcome or recanalisation was found [6].

In order to minimise OTT and optimise outcome, stroke pathways have evolved to allow the administration of bolus doses of alteplase as quickly as possible, sometimes in the CT or MRI scanner. The infusion may then be started after patients have been transferred to the acute stroke ward, emergency department, or clinical treatment areas resulting in delays between the administration of the bolus dose and commencement of the infusion. We observed longer bolus-infusion delays in patients with more severe strokes. This might be due to a longer time taken to transfer the patients from the CT table to the trolley which in our institution takes place before the infusion is commenced.

In healthy cohort and myocardial infarction studies using intravenous alteplase, the administration of a bolus dose immediately before commencement of infusion led to the attainment of steady state concentration in about 4 minutes [7] compared to about 20 minutes for infusion only dosing [2]. With a mean bolus-infusion delay of 9 minutes (approximately 2 half-lives) in our cohort, peak plasma concentrations after the administration of the bolus dose of alteplase would have significantly dropped by about 75% at the time of initiation of the infusion. Moreover, the infusion regimen delivers 1.5% of the total dose of alteplase per minute compared to 10% for the bolus if given over a minute. In cases of significant bolus-infusion delays, therefore, the contribution of the bolus injection towards steady state concentration could wane resulting in a delayed or suboptimum steady state concentration with potential knock-on effects on achieving optimum fibrinolysis. This is illustrated by delays in reaching initial peak concentrations of approximately 20 and 30 minutes in studies simulating bolus-infusion delays of 5 and 15 minutes, respectively [5].

Intuitively, a shorter bolus-infusion delay was postulated to be associated with improved outcome based on the attainment of near optimum plasma alteplase concentrations. The largely neutral three-month outcome measures may reflect the higher antiplatelet use and shorter mean OTT, which are associated with better outcome [1, 3] in the group with delays >8 minute albeit with a higher prethrombolysis median NIHSS (Table 1). It is possible that the effect of alteplase on the fibrinolytic system in AIS is not only defined by the optimum steady state concentrations envisaged from the immediate commencement of the infusion after the bolus. Other factors such as the speed of delivery of alteplase to, and saturation of, the plasminogen catalytic sites within the thrombus as evident in the effect of OTT on outcome after AIS, may also influence this [1]. This is consistent with the rationale for the departure from a complete reliance on infusions as employed in earlier trials [8].

Our study has a number of limitations. The data were retrospectively retrieved from a single centre with over 15-year experience of administering alteplase for AIS and it is possible that delays may vary in other centres.

## 5. Conclusions

Our study demonstrates that bolus-infusion delays are likely to be common. Delays of >8 minutes were not associated with significantly worse outcomes although the functional independence rate was lower and death rate was higher in this group. The series is however small to conclusively define the association between bolus-infusion delays and outcomes, as such larger prospective studies will be useful.

## Figures and Tables

**Table 1:** Demographic and clinical characteristics of patients. Data presented as mean [SD] for continuous variables and proportions (%) for categorical variables unless otherwise stated.

Characteristics	Bolus-infusion delays	
	0–8 minutes (*n* = 121)	>8 minutes (*n* = 108)
Male sex	50	56
Mean age (years)	73 [11]	72 [12]
Mean weight (kg)	72 [14]	76 [16]
Mean systolic blood pressure (mmHg)	144 [22]	142 [22]
Mean diastolic blood pressure (mmHg)	78 [15]	76 [15]
Mean admission blood glucose (mmol/L)	7 [2]	7 [3]
Hypertension	71	59
Diabetes mellitus	13	12
Atrial fibrillation	37	45
Congestive cardiac failure	8	4
Hyperlipidaemia	45	45
TIA/amaurosis fugax	10	6
Previous stroke	14	12
Peripheral vascular disease	6	5
Myocardial infarction or ischaemic heart disease	19	30
Current smoker	15	18
Ex-smoker	26	22
Antiplatelet use	53	60
Pre-stroke mRS, median (IQR)	1 (0–2)	1 (0–2)
Mean onset to treatment time (OTT) in minutes; proportions within groups		
0–90	79 [11]; 11%	74 [16]; 18%
91–180	132 [25]; 63%	132 [26]; 64%
181–270	212 [19]; 26%	209 [22]; 18%
0–270	147 [47]	136 [47]
Oxfordshire Community Stroke Project		
Total anterior circulatory stroke	41	56
Partial anterior circulatory stroke	45	35
Lacunar stroke	12	6
Posterior circulatory stroke	2	4
Pre-thrombolysis neurological severity per NIHSS score		
Median (IQR)*	10 [7–17]	14.5 [9–21]
Proportions within NIHSS categories (%)**		
0–9	49	30
10–19	35	37
>19	16	33

NIHSS—National Institutes of Health Stroke Scale; TIA—Transient Ischaemic Attack; mRS—modified Rankin Score. No significant differences between infusion delays of 0–8 and >8 minutes except for *(Mann Whitney U test, *P* = 0.016) and **(Pearson's *χ*2 test = 13.1, *P* = 0.001).
